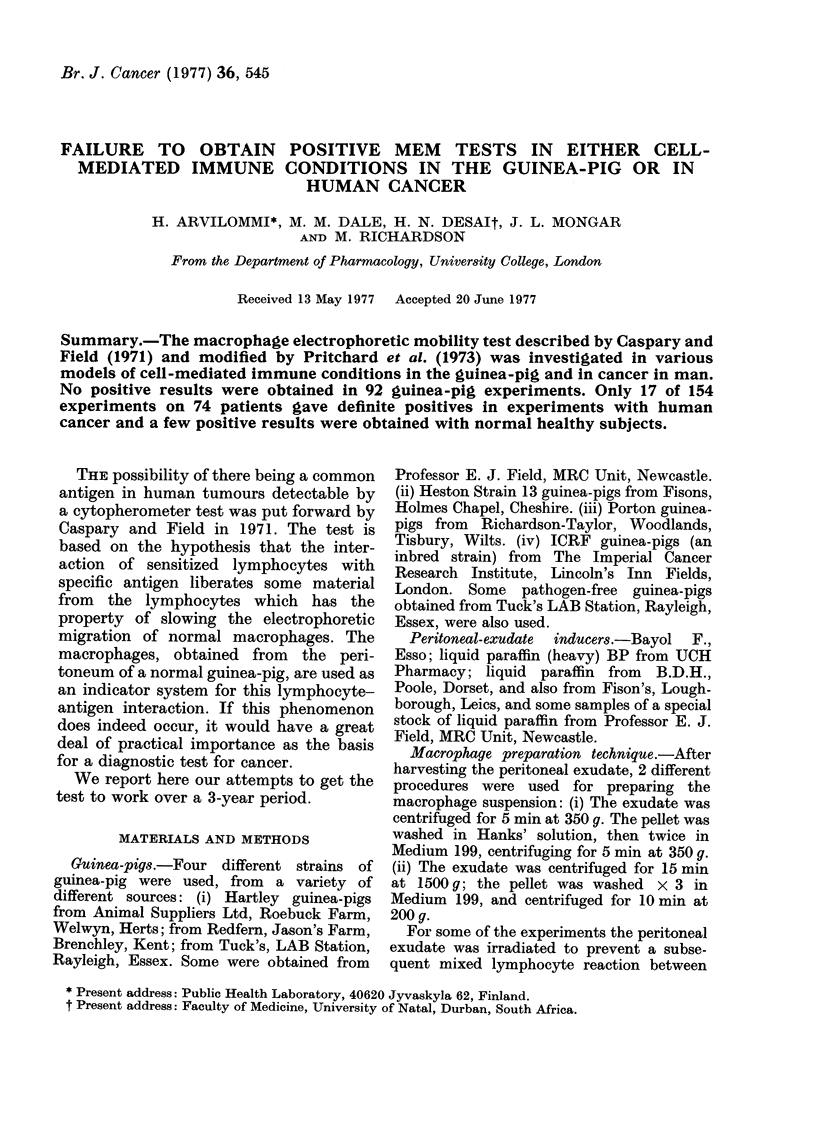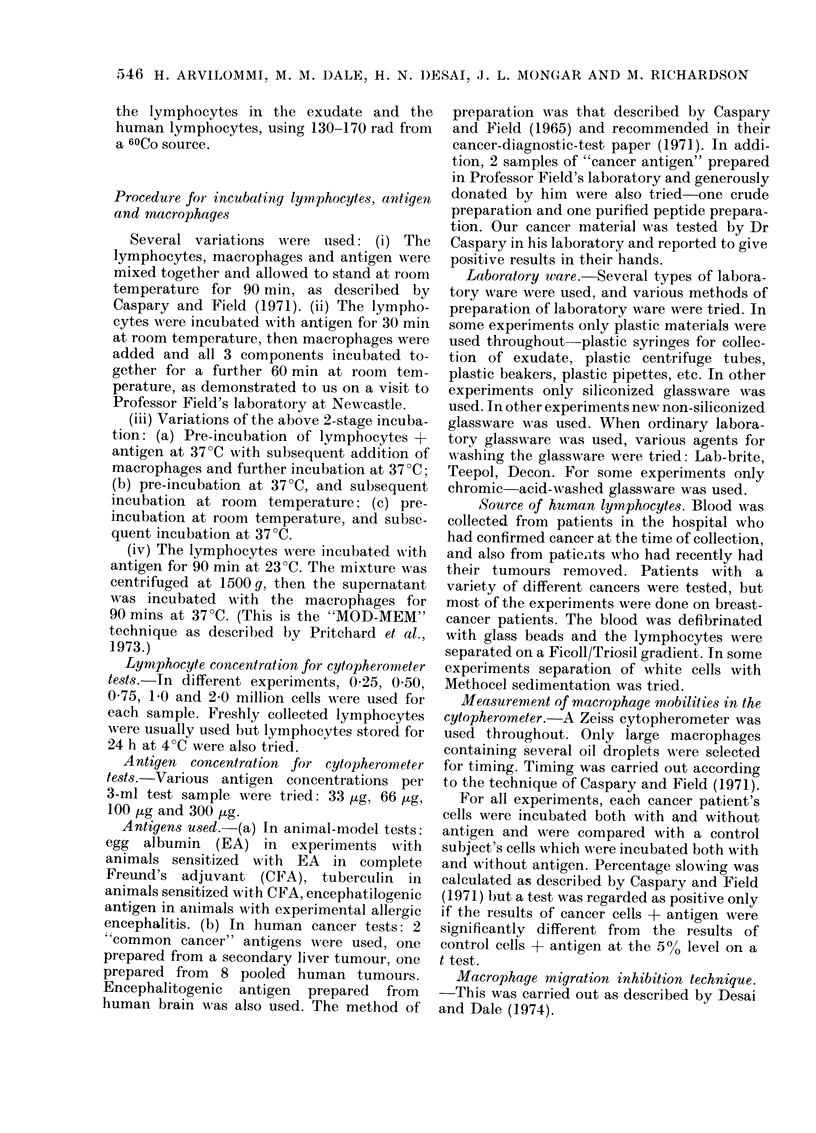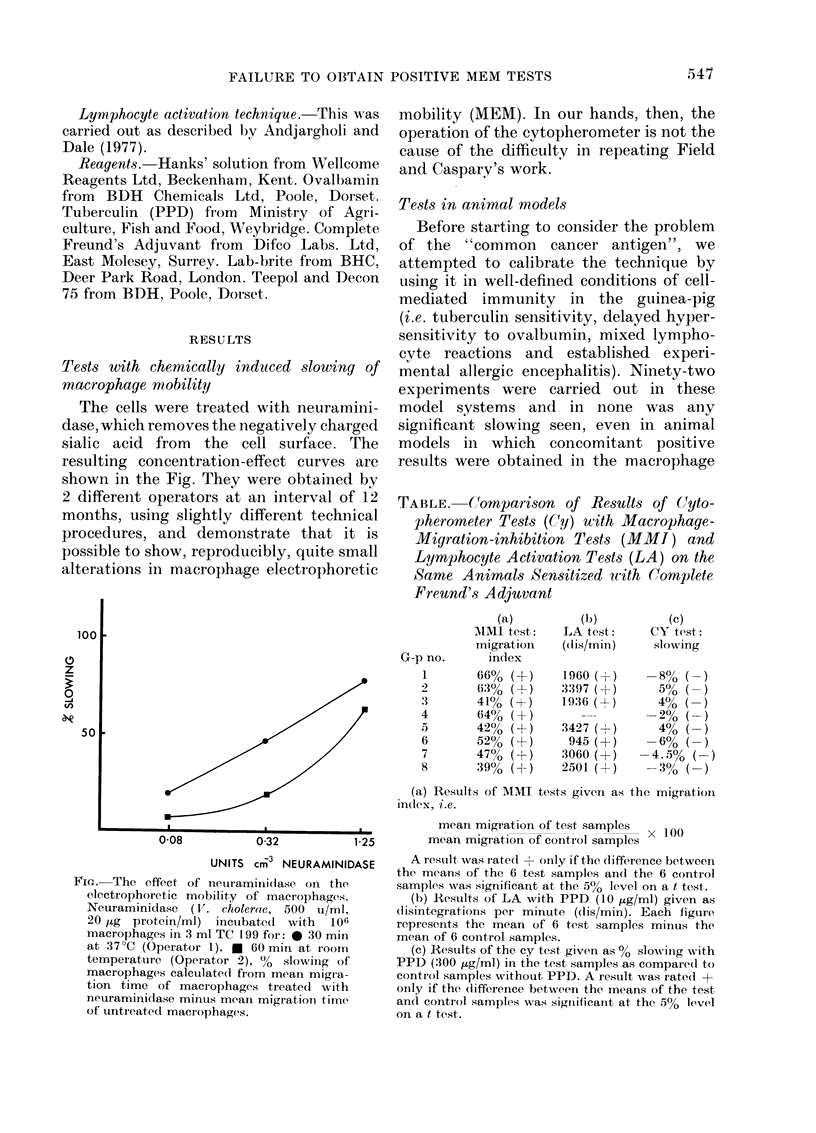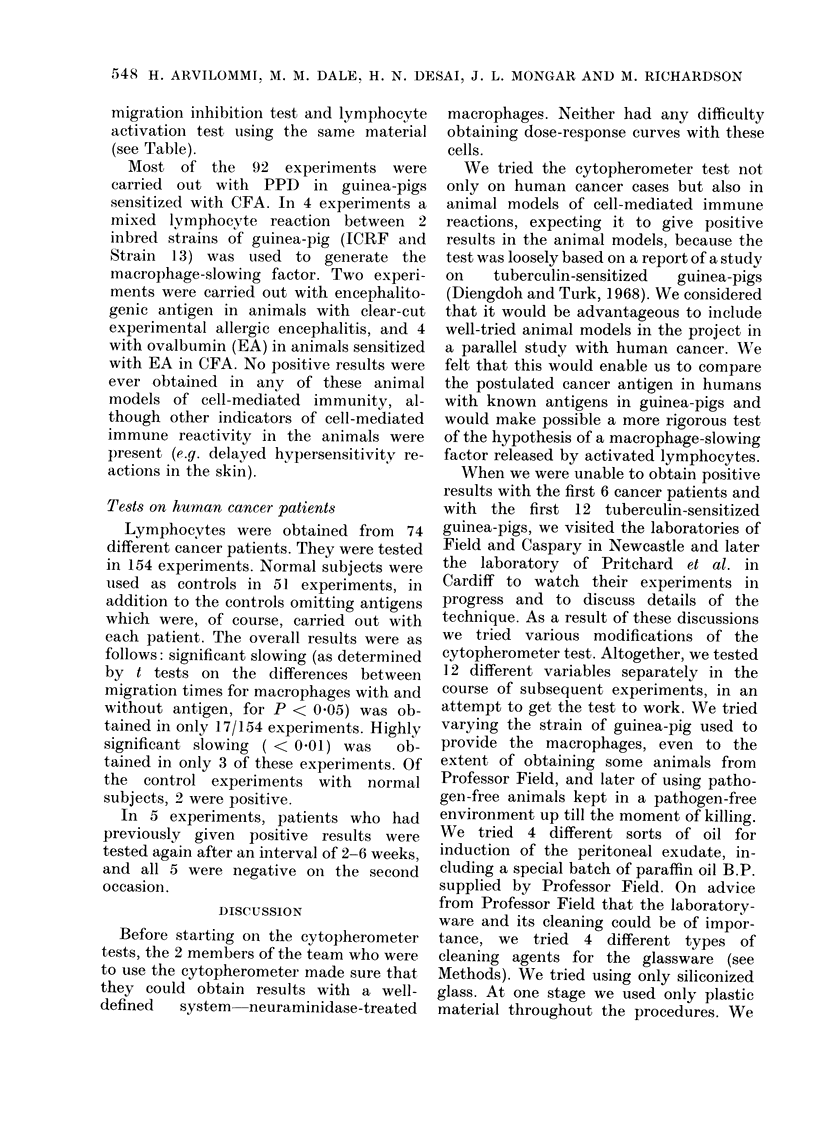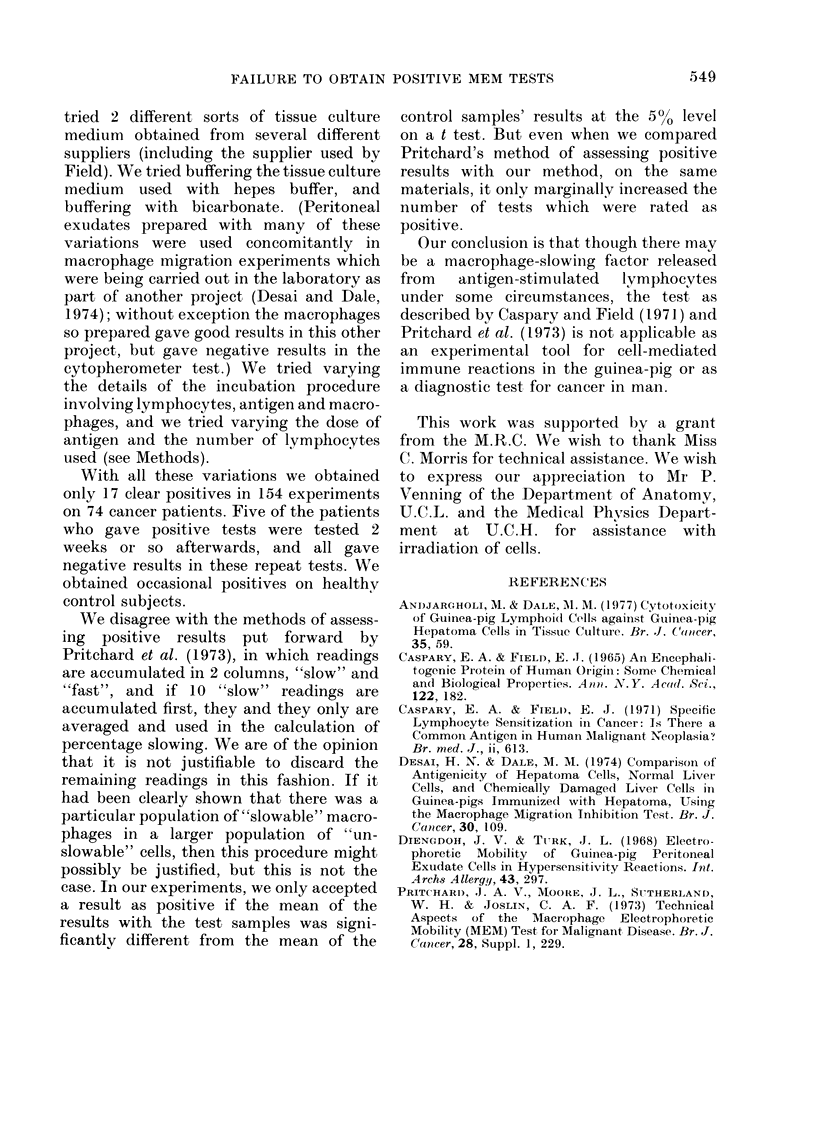# Failure to obtain positive MEM tests in either cell-mediated immune conditions in the guinea-pig or in human cancer.

**DOI:** 10.1038/bjc.1977.229

**Published:** 1977-11

**Authors:** H. Arvilommi, M. M. Dale, H. N. Desai, J. L. Mongar, M. Richardson

## Abstract

The macrophage electrophoretic mobility test described by Caspary and Field (1971) and modified by Pritchard et al. (1973) was investigated in various models of cell-mediated immune conditions in the guinea-pig and in cancer in man. No positive results were obtained in 92 guinea-pig experiments. Only 17 of 154 experiments on 74 patients gave definite positives in experiments with human cancer and a few positive results were obtained with normal healthy subjects.


Br. J. Cancer (1977) 36, 545

### FAILURE TO OBTAIN POSITIVE MEM TESTS IN EITHER CELL-MEDIATED IMMUNE CONDITIONS IN THE GUINEA-PIG OR IN HUMAN CANCER

H. ARVILOMMI*, M. M. DALE, H. N. DESAI†, J. L. MONGAR AND M. RICHARDSON

*From the Department of Pharmacology, University College, London*

Received 13 May 1977 Accepted 20 June 1977

**Summary.—The macrophage electrophoretic mobility test described by Caspary and Field (1971) and modified by Pritchard *et al.* (1973) was investigated in various models of cell-mediated immune conditions in the guinea-pig and in cancer in man. No positive results were obtained in 92 guinea-pig experiments. Only 17 of 154 experiments on 74 patients gave definite positives in experiments with human cancer and a few positive results were obtained with normal healthy subjects.**

THE possibility of there being a common antigen in human tumours detectable by a cytopherometer test was put forward by Caspary and Field in 1971. The test is based on the hypothesis that the interaction of sensitized lymphocytes with specific antigen liberates some material from the lymphocytes which has the property of slowing the electrophoretic migration of normal macrophages. The macrophages, obtained from the peritoneum of a normal guinea-pig, are used as an indicator system for this lymphocyte–antigen interaction. If this phenomenon does indeed occur, it would have a great deal of practical importance as the basis for a diagnostic test for cancer.

We report here our attempts to get the test to work over a 3-year period.

### MATERIALS AND METHODS

*Guinea-pigs.*—Four different strains of guinea-pig were used, from a variety of different sources: (i) Hartley guinea-pigs from Animal Suppliers Ltd, Roebuck Farm, Welwyn, Herts; from Redfern, Jason's Farm, Brenchley, Kent; from Tuck's, LAB Station, Rayleigh, Essex. Some were obtained from Professor E. J. Field, MRC Unit, Newcastle. (ii) Heston Strain 13 guinea-pigs from Fisons, Holmes Chapel, Cheshire. (iii) Porton guinea-pigs from Richardson-Taylor, Woodlands, Tisbury, Wilts. (iv) ICRF guinea-pigs (an inbred strain) from The Imperial Cancer Research Institute, Lincoln's Inn Fields, London. Some pathogen-free guinea-pigs obtained from Tuck's LAB Station, Rayleigh, Essex, were also used.

*Peritoneal-exudate inducers.*—Bayol F., Esso; liquid paraffin (heavy) BP from UCH Pharmacy; liquid paraffin from B.D.H., Poole, Dorset, and also from Fison's, Loughborough, Leics, and some samples of a special stock of liquid paraffin from Professor E. J. Field, MRC Unit, Newcastle.

*Macrophage preparation technique.*—After harvesting the peritoneal exudate, 2 different procedures were used for preparing the macrophage suspension: (i) The exudate was centrifuged for 5 min at 350 $g$. The pellet was washed in Hanks' solution, then twice in Medium 199, centrifuging for 5 min at 350 $g$. (ii) The exudate was centrifuged for 15 min at 1500 $g$; the pellet was washed $\times$ 3 in Medium 199, and centrifuged for 10 min at 200 $g$.

For some of the experiments the peritoneal exudate was irradiated to prevent a subsequent mixed lymphocyte reaction between

* Present address: Public Health Laboratory, 40620 Jyvaskyla 62, Finland.
† Present address: Faculty of Medicine, University of Natal, Durban, South Africa.

the lymphocytes in the exudate and the human lymphocytes, using 130–170 rad from a $^{60}$Co source.

*Procedure for incubating lymphocytes, antigen and macrophages*

Several variations were used: (i) The lymphocytes, macrophages and antigen were mixed together and allowed to stand at room temperature for 90 min, as described by Caspary and Field (1971). (ii) The lymphocytes were incubated with antigen for 30 min at room temperature, then macrophages were added and all 3 components incubated together for a further 60 min at room temperature, as demonstrated to us on a visit to Professor Field's laboratory at Newcastle.

(iii) Variations of the above 2-stage incubation: (a) Pre-incubation of lymphocytes + antigen at 37°C with subsequent addition of macrophages and further incubation at 37°C; (b) pre-incubation at 37°C, and subsequent incubation at room temperature; (c) pre-incubation at room temperature, and subsequent incubation at 37°C.

(iv) The lymphocytes were incubated with antigen for 90 min at 23°C. The mixture was centrifuged at 1500 *g*, then the supernatant was incubated with the macrophages for 90 mins at 37°C. (This is the "MOD-MEM" technique as described by Pritchard *et al.*, 1973.)

*Lymphocyte concentration for cytopherometer tests.*—In different experiments, 0·25, 0·50, 0·75, 1·0 and 2·0 million cells were used for each sample. Freshly collected lymphocytes were usually used but lymphocytes stored for 24 h at 4°C were also tried.

*Antigen concentration for cytopherometer tests.*—Various antigen concentrations per 3-ml test sample were tried: 33 μg, 66 μg, 100 μg and 300 μg.

*Antigens used.*—(a) In animal-model tests: egg albumin (EA) in experiments with animals sensitized with EA in complete Freund's adjuvant (CFA), tuberculin in animals sensitized with CFA, encephatilogenic antigen in animals with experimental allergic encephalitis. (b) In human cancer tests: 2 "common cancer" antigens were used, one prepared from a secondary liver tumour, one prepared from 8 pooled human tumours. Encephalitogenic antigen prepared from human brain was also used. The method of preparation was that described by Caspary and Field (1965) and recommended in their cancer-diagnostic-test paper (1971). In addition, 2 samples of "cancer antigen" prepared in Professor Field's laboratory and generously donated by him were also tried—one crude preparation and one purified peptide preparation. Our cancer material was tested by Dr Caspary in his laboratory and reported to give positive results in their hands.

*Laboratory ware.*—Several types of laboratory ware were used, and various methods of preparation of laboratory ware were tried. In some experiments only plastic materials were used throughout—plastic syringes for collection of exudate, plastic centrifuge tubes, plastic beakers, plastic pipettes, etc. In other experiments only siliconized glassware was used. In other experiments new non-siliconized glassware was used. When ordinary laboratory glassware was used, various agents for washing the glassware were tried: Lab-brite, Teepol, Decon. For some experiments only chromic—acid-washed glassware was used.

*Source of human lymphocytes.* Blood was collected from patients in the hospital who had confirmed cancer at the time of collection, and also from patients who had recently had their tumours removed. Patients with a variety of different cancers were tested, but most of the experiments were done on breast-cancer patients. The blood was defibrinated with glass beads and the lymphocytes were separated on a Ficoll/Triosil gradient. In some experiments separation of white cells with Methocel sedimentation was tried.

*Measurement of macrophage mobilities in the cytopherometer.*—A Zeiss cytopherometer was used throughout. Only large macrophages containing several oil droplets were selected for timing. Timing was carried out according to the technique of Caspary and Field (1971).

For all experiments, each cancer patient's cells were incubated both with and without antigen and were compared with a control subject's cells which were incubated both with and without antigen. Percentage slowing was calculated as described by Caspary and Field (1971) but a test was regarded as positive only if the results of cancer cells + antigen were significantly different from the results of control cells + antigen at the 5% level on a *t* test.

*Macrophage migration inhibition technique.*—This was carried out as described by Desai and Dale (1974).

*Lymphocyte activation technique.*—This was carried out as described by Andjargholi and Dale (1977).

*Reagents.*—Hanks' solution from Wellcome Reagents Ltd, Beckenham, Kent. Ovalbamin from BDH Chemicals Ltd, Poole, Dorset. Tuberculin (PPD) from Ministry of Agriculture, Fish and Food, Weybridge. Complete Freund's Adjuvant from Difco Labs. Ltd, East Molesey, Surrey. Lab-brite from BHC, Deer Park Road, London. Teepol and Decon 75 from BDH, Poole, Dorset.

### RESULTS

### *Tests with chemically induced slowing of macrophage mobility*

The cells were treated with neuraminidase, which removes the negatively charged sialic acid from the cell surface. The resulting concentration-effect curves are shown in the Fig. They were obtained by 2 different operators at an interval of 12 months, using slightly different technical procedures, and demonstrate that it is possible to show, reproducibly, quite small alterations in macrophage electrophoretic mobility (MEM). In our hands, then, the operation of the cytopherometer is not the cause of the difficulty in repeating Field and Caspary's work.

FIG.—The effect of neuraminidase on the electrophoretic mobility of macrophages. Neuraminidase (*V. cholerae*, 500 u/ml, 20 μg protein/ml) incubated with $10^6$ macrophages in 3 ml TC 199 for: ● 30 min at 37°C (Operator 1). ■ 60 min at room temperature (Operator 2), % slowing of macrophages calculated from mean migration time of macrophages treated with neuraminidase minus mean migration time of untreated macrophages.

### *Tests in animal models*

Before starting to consider the problem of the "common cancer antigen", we attempted to calibrate the technique by using it in well-defined conditions of cell-mediated immunity in the guinea-pig (*i.e.* tuberculin sensitivity, delayed hypersensitivity to ovalbumin, mixed lymphocyte reactions and established experimental allergic encephalitis). Ninety-two experiments were carried out in these model systems and in none was any significant slowing seen, even in animal models in which concomitant positive results were obtained in the macrophage

TABLE.—*Comparison of Results of Cytopherometer Tests (Cy) with Macrophage-Migration-inhibition Tests (MMI) and Lymphocyte Activation Tests (LA) on the Same Animals Sensitized with Complete Freund's Adjuvant*

| G-p no. | (a) MMI test: migration index | (b) LA test: (dis/min) | (c) CY test: slowing |
|---|---|---|---|
| 1 | 66% (+) | 1960 (+) | −8% (−) |
| 2 | 63% (+) | 3397 (+) | 5% (−) |
| 3 | 41% (+) | 1936 (+) | 4% (−) |
| 4 | 64% (+) | — | −2% (−) |
| 5 | 42% (+) | 3427 (+) | 4% (−) |
| 6 | 52% (+) | 945 (+) | −6% (−) |
| 7 | 47% (+) | 3060 (+) | −4.5% (−) |
| 8 | 39% (+) | 2501 (+) | −3% (−) |

(a) Results of MMI tests given as the migration index, *i.e.*

$$\frac{\text{mean migration of test samples}}{\text{mean migration of control samples}} \times 100$$

A result was rated + only if the difference between the means of the 6 test samples and the 6 control samples was significant at the 5% level on a *t* test.

(b) Results of LA with PPD (10 μg/ml) given as disintegrations per minute (dis/min). Each figure represents the mean of 6 test samples minus the mean of 6 control samples.

(c) Results of the cy test given as % slowing with PPD (300 μg/ml) in the test samples as compared to control samples without PPD. A result was rated + only if the difference between the means of the test and control samples was significant at the 5% level on a *t* test.

migration inhibition test and lymphocyte activation test using the same material (see Table).

Most of the 92 experiments were carried out with PPD in guinea-pigs sensitized with CFA. In 4 experiments a mixed lymphocyte reaction between 2 inbred strains of guinea-pig (ICRF and Strain 13) was used to generate the macrophage-slowing factor. Two experiments were carried out with encephalitogenic antigen in animals with clear-cut experimental allergic encephalitis, and 4 with ovalbumin (EA) in animals sensitized with EA in CFA. No positive results were ever obtained in any of these animal models of cell-mediated immunity, although other indicators of cell-mediated immune reactivity in the animals were present (*e.g.* delayed hypersensitivity reactions in the skin).

*Tests on human cancer patients*

Lymphocytes were obtained from 74 different cancer patients. They were tested in 154 experiments. Normal subjects were used as controls in 51 experiments, in addition to the controls omitting antigens which were, of course, carried out with each patient. The overall results were as follows: significant slowing (as determined by $t$ tests on the differences between migration times for macrophages with and without antigen, for $P < 0{\cdot}05$) was obtained in only 17/154 experiments. Highly significant slowing ($< 0{\cdot}01$) was obtained in only 3 of these experiments. Of the control experiments with normal subjects, 2 were positive.

In 5 experiments, patients who had previously given positive results were tested again after an interval of 2–6 weeks, and all 5 were negative on the second occasion.

### DISCUSSION

Before starting on the cytopherometer tests, the 2 members of the team who were to use the cytopherometer made sure that they could obtain results with a well-defined system—neuraminidase-treated macrophages. Neither had any difficulty obtaining dose-response curves with these cells.

We tried the cytopherometer test not only on human cancer cases but also in animal models of cell-mediated immune reactions, expecting it to give positive results in the animal models, because the test was loosely based on a report of a study on tuberculin-sensitized guinea-pigs (Diengdoh and Turk, 1968). We considered that it would be advantageous to include well-tried animal models in the project in a parallel study with human cancer. We felt that this would enable us to compare the postulated cancer antigen in humans with known antigens in guinea-pigs and would make possible a more rigorous test of the hypothesis of a macrophage-slowing factor released by activated lymphocytes.

When we were unable to obtain positive results with the first 6 cancer patients and with the first 12 tuberculin-sensitized guinea-pigs, we visited the laboratories of Field and Caspary in Newcastle and later the laboratory of Pritchard *et al.* in Cardiff to watch their experiments in progress and to discuss details of the technique. As a result of these discussions we tried various modifications of the cytopherometer test. Altogether, we tested 12 different variables separately in the course of subsequent experiments, in an attempt to get the test to work. We tried varying the strain of guinea-pig used to provide the macrophages, even to the extent of obtaining some animals from Professor Field, and later of using pathogen-free animals kept in a pathogen-free environment up till the moment of killing. We tried 4 different sorts of oil for induction of the peritoneal exudate, including a special batch of paraffin oil B.P. supplied by Professor Field. On advice from Professor Field that the laboratory-ware and its cleaning could be of importance, we tried 4 different types of cleaning agents for the glassware (see Methods). We tried using only siliconized glass. At one stage we used only plastic material throughout the procedures. We tried 2 different sorts of tissue culture medium obtained from several different suppliers (including the supplier used by Field). We tried buffering the tissue culture medium used with hepes buffer, and buffering with bicarbonate. (Peritoneal exudates prepared with many of these variations were used concomitantly in macrophage migration experiments which were being carried out in the laboratory as part of another project (Desai and Dale, 1974); without exception the macrophages so prepared gave good results in this other project, but gave negative results in the cytopherometer test.) We tried varying the details of the incubation procedure involving lymphocytes, antigen and macrophages, and we tried varying the dose of antigen and the number of lymphocytes used (see Methods).

With all these variations we obtained only 17 clear positives in 154 experiments on 74 cancer patients. Five of the patients who gave positive tests were tested 2 weeks or so afterwards, and all gave negative results in these repeat tests. We obtained occasional positives on healthy control subjects.

We disagree with the methods of assessing positive results put forward by Pritchard *et al.* (1973), in which readings are accumulated in 2 columns, "slow" and "fast", and if 10 "slow" readings are accumulated first, they and they only are averaged and used in the calculation of percentage slowing. We are of the opinion that it is not justifiable to discard the remaining readings in this fashion. If it had been clearly shown that there was a particular population of "slowable" macrophages in a larger population of "unslowable" cells, then this procedure might possibly be justified, but this is not the case. In our experiments, we only accepted a result as positive if the mean of the results with the test samples was significantly different from the mean of the control samples' results at the 5% level on a $t$ test. But even when we compared Pritchard's method of assessing positive results with our method, on the same materials, it only marginally increased the number of tests which were rated as positive.

Our conclusion is that though there may be a macrophage-slowing factor released from antigen-stimulated lymphocytes under some circumstances, the test as described by Caspary and Field (1971) and Pritchard *et al.* (1973) is not applicable as an experimental tool for cell-mediated immune reactions in the guinea-pig or as a diagnostic test for cancer in man.

This work was supported by a grant from the M.R.C. We wish to thank Miss C. Morris for technical assistance. We wish to express our appreciation to Mr P. Venning of the Department of Anatomy, U.C.L. and the Medical Physics Department at U.C.H. for assistance with irradiation of cells.

### REFERENCES

ANDJARGHOLI, M. & DALE, M. M. (1977) Cytotoxicity of Guinea-pig Lymphoid Cells against Guinea-pig Hepatoma Cells in Tissue Culture. *Br. J. Cancer*, **35**, 59.

CASPARY, E. A. & FIELD, E. J. (1965) An Encephalitogenic Protein of Human Origin: Some Chemical and Biological Properties. *Ann. N.Y. Acad. Sci.*, **122**, 182.

CASPARY, E. A. & FIELD, E. J. (1971) Specific Lymphocyte Sensitization in Cancer: Is There a Common Antigen in Human Malignant Neoplasia? *Br. med. J.*, ii, 613.

DESAI, H. N. & DALE, M. M. (1974) Comparison of Antigenicity of Hepatoma Cells, Normal Liver Cells, and Chemically Damaged Liver Cells in Guinea-pigs Immunized with Hepatoma, Using the Macrophage Migration Inhibition Test. *Br. J. Cancer*, **30**, 109.

DIENGDOH, J. V. & TURK, J. L. (1968) Electrophoretic Mobility of Guinea-pig Peritoneal Exudate Cells in Hypersensitivity Reactions. *Int. Archs Allergy*, **43**, 297.

PRITCHARD, J. A. V., MOORE, J. L., SUTHERLAND, W. H. & JOSLIN, C. A. F. (1973) Technical Aspects of the Macrophage Electrophoretic Mobility (MEM) Test for Malignant Disease. *Br. J. Cancer*, **28**, Suppl. 1, 229.